# Clinical pharmacology of tyrosine kinase inhibitors becoming generic drugs: the regulatory perspective

**DOI:** 10.1186/1756-9966-33-15

**Published:** 2014-02-07

**Authors:** Niels Eckstein, Lea Röper, Bodo Haas, Henrike Potthast, Ulrike Hermes, Christoph Unkrig, Frauke Naumann-Winter, Harald Enzmann

**Affiliations:** 1 Federal Institute of Drugs and Medical Devices, Kurt-Georg-Kiesinger-Allee 3, Bonn 53175, Germany

**Keywords:** Narrow therapeutic index drugs (NTID), Tyrosine kinase inhibitors (TKI), Orphan drug status

## Abstract

Over the last decades, billions have been spent and huge efforts have been taken in basic and clinical cancer research [*CA Cancer J Clin* ***63:*** *11-30*]. About a decade ago, the arms race between drugs and cancer cells reached a new level by introduction of tyrosine kinase inhibitors (TKI) into pharmacological anti-cancer therapy. According to their molecular mechanism of action, TKI in contrast to so-called “classic” or “conventional” cytostatics belong to the group of targeted cancer medicines, characterized by accurately fitting with biological structures (i.e. active centers of kinases). Numerous (partly orphan) indications are covered by this new class of substances. Approximately ten years after the first substances of this class of medicines were authorized, patent protection will end within the next years. The following article covers clinical meaning and regulatory status of anti-cancer TKI and gives an outlook to what is expected from the introduction of generic anti-cancer TKI.

## Introduction

Pharmacological cancer therapy for decades was performed with non-targeted mostly DNA-interacting cytostatic drugs. Administration of these so-called conventional cytostatics usually is entailed with severe side-effects [1]. One of the main disadvantages of those substances is that they do not specifically target cancer cells but all (also benign) rapidly dividing cells. This non-specific mechanism of action was the rationale to develop specifically targeted anti-cancer TKI. Initially, great expectations were associated with these drugs; some were met, others not. Tyrosine kinase inhibitors (TKI) are a very worthy additional option for physicians in clinical management of certain types and lines of cancer treatment (refer to Table 1 for a tabular overview). However, the initial expectation of a new era of cancer-therapy with substantially less side effects was not fulfilled. TKI have numerous, partly severe side effects eventually entailed with fatal outcome (Table 2). On the other hand, when a tumor becomes resistant to conventional or targeted anti-cancer therapy, TKI serve as additional options in second-, third- and/or fourth-line therapy regimes according to their approved indications. For instance Sunitinib is approved after Imatinib resistance formation in gastrointestinal stromal tumors (GIST), and Lapatinib after non-responding to antracycline- or taxane-based chemotherapy in combination with Trastuzumab in HER-2 positive breast cancer. Taken together, TKI are a valuable extension of the cancer drug armamentarium [2,3].

**Table 1:** General information on anti-cancer TKI

**Tyrosine kinase inhibitor (INN)**	**Branded name**	**Market Authorization Holder (MAH)**	**Target tyrosine kinases**	**Indication(s)**	**European birth date**	**CMA**	**Orphan designation**
Bosutinib	Bosulif®	Pfizer	BCR-ABL,SRC	Patients with CML for which Imatinib, Nilotinib, and Dasatinib are not appropriate	27^th^ March 2013	Yes	CML
Dasatinib	Sprycel®	Bristol-Myers Squibb	BCR-ABL	CML	23^th^ December 2005	No	CML, ALL
Erlotinib	Tarceva®	Hoffman-La Roche	EGFR	NSCLC, pancreatic cancer	19^th^ September 2005	No	No
Gefitinib	Iressa®	Astra Zeneca	EGFR	NSCLC in carriers of activating EGFR-mutations	24^th^ June 2010	No	No
Imatinib	Glivec®	Novartis	BCR-ABL, KIT, PDGFR-A, PDGFR-B	CML, GIST, BCR-ABL- positive ALL, dermatofibrosarcoma protuberans, myeloproliferative neoplasms, hypereosinophilic syndromes	7^th^ of November 2001	No	Expired and withdrawn
Lapatinib	Tyverb®	Glaxo Smith Kline	ERBB2 (HER-2)	HER-2 positive breast cancer	10^th^ June 2008	Yes	No
Nilotinib^1^	Tasigna®	Novartis	BCR-ABL, KIT,PDGFR-A, PDGFR-B	CML	19^th^ November 2007	No	CML
Pazopanib	Votrient®	Glaxo Smith Kline	VEGFR, PDGFR, KIT	Renal cell carcinoma, STS	14^th^ June 2010	No	Withdrawn
Ponatinib^2^	Iclusig®	Ariad	BCR-ABL	Patients with CML for which Imatinib, Nilotinib, and Dasatinib are not appropriate (or patients carrying a T315I single-point-mutation)	1^st^ July 2013		CML, ALL
Sorafenib	Nexavar®	Bayer	VEGFR-2,VEGFR-3	Renal cell carcinoma, hepatocellular carcinoma	19^th^ July 2006	No	Renal cell carcinoma, Hepatocellular carcinoma
Sunitinib	Sutent®	Pfizer	VEGFR 1-3, PDGFR-A, PDGFR-B; KIT, FLT3	Renal cell carcinoma, GIST, pNET	19^th^ July 2006	Initially, then full approval	Withdrawn

ALL, acute lymphatic leukemia; CML, chronic myeloid leukemia ; CMA, Conditional Marketing Authorization (none of the above mentioned is currently authorized under exceptional circumstances, according to European Medicines Agency (EMA) website accessed in Sept 2013 [15]); GIST, gastrointestinal stromal tumor; MA, Marketing Authorization; MAH, Marketing Authorization Holder; NSCLC, non-small cell lung cancer; pNET, pancreatic neuroendocrine tumors; STS, soft tissue sarcoma; ^1^Nilotinib is similar to Imatinib according to the orphan regulation; ^2^US-Food and Drug Administration (FDA) asked the manufacturer of Ponatinib to suspend marketing due to the risk of life-threatening blood clots and severe narrowing of blood vessels; source of information: European Public Assessment Reports (EPARs) of the above mentioned TKI [15].

**Table 2:** Safety profiles of TKI

**Small molecule TKI**	**CNS**	**Nerve disorders**	**Eye disorders**	**Heart disorders**	**Lung airways disorders**	**Thyroid disorders**	**Liver, Bile disorders**
Bosutinib		XX		XX	XX		XX
Dasatinib	X	XX	XX	XX	XX		X
Erlotinib	X	XX	XX		XX		X
Gefitinib			XX		XX		XX
Imatinib	X	XX	XX	X	XX	X	XX
Lapatinib	X	XX		X	XX		XX
Nilotinib	X	XX	XX	XX	XX		XX
Pazopanib		XX	XX	X	XX	XX	XX
Ponatinib		XX	XX	XX	XX		XX
Sorafenib	X	XX		X	X		X
Sunitinib	X	XX	XX	X	XX	XX	X
**Small molecule TKI**	**Gastrointestinal disorders**	**Renal disorders**	**Musculoskeletal and bone disorders**	**Blood and lymphatic system**	**Vascular disorders**	**Skin disorders**	**CMR**
Bosutinib	XX	XX	XX	XX		XX	
Dasatinib	XX	X	X	XX	XX	XX	XX
Erlotinib	XX	XX		X		XX	XX
Gefitinib	XX	XX			XX	XX	XX
Imatinib	XX	X	XX	XX	X	XX	XX
Lapatinib	XX		XX		XX	XX	XX
Nilotinib	X	X	X	XX	X	XX	XX
Pazopanib	XX	XX	XX	XX	XX	XX	XX
Ponatinib	XX		XX	XX	XX	XX	
Sorafenib	X	X	X	XX	XX	XX	XX
Sunitinib	XX	XX	XX	XX	XX	XX	XX

XX = common, very common; X = rare, uncommon; CMR, carcinogenic, mutagenic and toxic for reproductive system; CNS, central nervous system; source of information: Summaries of Product Characteristics (SmPCs) of marketed TKI [16].

### Molecular mechanism of action

Many chemotherapy-naive and nearly all drug resistant tumors are characterized by pronounced Receptor-Tyrosine-Kinase (RTK) signaling. This pattern is at least in part due to the fact that chemoresistance can be triggered by overexpression and/or activation of RTKs: ERB B1-4, IGF-1R, VEGFR 1-3, and PDGF-receptor family members [4,5]. The underlying mechanisms of this over-activation are diverse and comprise at least the following mechanisms [6].

→ Formation of a self-sustaining autocrine loop with secreted growth factors such as EGF, VEGF, PDGF, amphiregulin or others [5].

→ Expression of intrinsically active RTK in the cell membrane [7].

→ Over-activation of downstream signaling by imbalance of tumor-suppressor genes (p53, PTEN) and (proto-) oncogenes (PI3K, monomeric G Proteins such as RAS, RAF and others) [8] etc.

**Figure 1:**
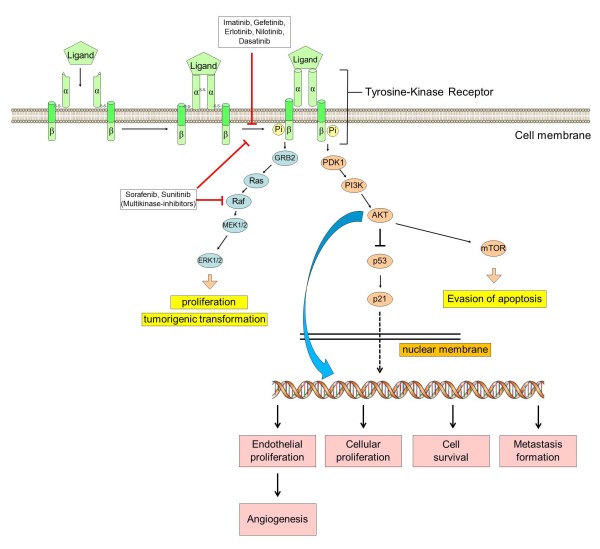
Schematic model of tumorigenic signaling pathways and their inhibition by anti-cancer-TKI.

*In vitro* investigations of cancer cell-lines derived from numerous tumor-entities regularly uncovered receptor tyrosine kinase (i.e. EGFR) activation by phosphorylation of specific residues located in the β-subunit [9,10]. Downstream the adaptor protein GAB1 (Grb2-associated binder 1) recruits PI3 kinase to phosphorylated EGFR [11]. The main function of GAB1 is to enhance PI3K/AKT activation thereby prolonging MAPK signaling [12]. While RAS/RAF/MEK/ERK signaling cascade usually ends up in cellular proliferation and tumorigenic transformation, enhanced AKT-kinase signaling usually is entailed with evasion of apoptosis, which is the turning-point in drug resistance formation [13]. Given this, TKI can interrupt signaling cascades evading apoptosis, thereby re-sensitizing cancer cells to induction of apoptosis. Figure 1 gives a schematic overview of the molecular mechanisms of action of TKI.

### Challenges of generic TKI drugs in cancer therapy

According to their European Birth Date during the past decade, these substances successively will be running off-patent within the next years (Table 1). From a regulatory point of view, this raises the question how marketing authorization applications (MAA) should be filed and especially, how therapeutic equivalence should be established for generic applications. In general, demonstrated bioequivalence (BE) allows generic medicinal products to refer to the efficacy and safety data of the originator medicinal product. It is easy to anticipate, that numerous questions in this regard will arise in the near future.

Aqueous (non-complicated) intravenously applied drug products have a 100% bioavailability directly per definition, thus, no BE studies are required for a MAA of such generic drugs. However, for orally applied drug products, BE with the originator product needs to be shown, which may be done using patients or healthy volunteers in respective in vivo studies or by means of comparative in-vitro investigations.

Since decades BE-acceptance criteria for AUC and C_max_ require the 90% confidence intervals being completely within 80 - 125% (for AUC and C_max_) to assume BE. The acceptance range may be tightened to 90 - 111% for one or both pharmacokinetic characteristics according to the European BE-Guideline [14] in the case of narrow therapeutic index drugs (NTID). In cases of class I and III compounds having identified not to have a narrow therapeutic index – specific in-vitro dissolution data may substitute for human BE-studies considering also particular requirements on excipients. This concept follows the principles of the biopharmaceutical classification system (BCS) [14].

It is likely that numerous questions in regard to the appropriate data package will arise in the near future including questions on the appropriate study design, on the appropriate study population, nutrition status, single or repeated dose-design, appropriate BCS classification of the individual compound or the classification as NTID.

MAA for new generics may be processed via different regulatory authorizations routes, i.e. national procedures in European member states, decentralized procedures involving several European member states or centralized procedures for all European member states. As the latter is an option only for generics for which the originator medicinal products already obtained marketing authorization from a centralized procedure, this option may receive more attention with the increasing number of medicinal products with centralized authorizations that are running off data protection and patent in the next years.

With the intent to enable a consistent approach for these different routes the European Medicines Agency (EMA) issued an initiative to harmonize the data requirements throughout European Member States, i.e. EMA initiated a pro-active program “Product-specific Bioequivalence-Guidance for Generics” [15]. EMA defines the objective of this initiative as follows: “Product specific guidance for the bioequivalence assessment of immediate release generic formulations should *a priori* be defined.” Thus, applicants should be given a clear scientific guidance, how to design BE-studies and, thus, how to file generic applications. This program includes BCS-classifications for drug substances, so that a harmonized view on the BCS classification and consequently the appropriateness of a BCS-based biowaiver approach can be expected for respective products. Furthermore, the guidance provides information on the type of expected data, e.g. appropriate study population (patients or healthy volunteers), mode of administration (fasten or fed), single dose or steady state-design, appropriate dose strength and analytes, the classification as NTID. The first wave of 16 medicinal products is dominated by anti-infectives and TKI. Dasatinib, Erlotinib, Imatinib, Sorafenib and Sunitinib are covered in this first round of harmonization [15].

From a clinician’s point of view regarding drug safety (Table 2), one could be tempted to assume that all anti-cancer medicinal products including TKI are considered as NTID. However, this is not the case. Different definitions of NTID by different regulatory agencies do exist. US-FDA classification of **narrow therapeutic ratio**:

→ Less than a 2-fold difference in median lethal dose (LD_50_) and median effective dose values (ED_50_), -or

→ Less than 2-fold difference in the minimum toxic concentrations (MTC) and minimum effective concentrations (MEC) in the blood or

→ Safe and effective use of the drug products require careful titration and patient monitoring.

In contrast to the US, for the EU no list of substances with NTID-designation is available. So far the consideration of a given substance as NTID is mainly based on national traditions. Only for a few medicinal substances (e.g. Ciclosporine, Tacrolimus) a harmonized EU decision was issued by a referral procedure. According to the draft “Product-specific Bioequivalence - Guidance for Generics” no drug is newly considered as NTID, only Tacrolimus is considered as such based on the previously finalized referral procedure.

According to the European BE-Guideline [14] clinical considerations are the basis for NTID decisions. Thus, safety-and efficacy profile have to be taken into account.

Most conventional cytotoxic medicinal products are given parenterally for a short duration in repeated cycles. They are mostly dosed on an individual basis (e.g. body surface or weight). The recommended dose is normally the maximum tolerated dose (MTD) or close to it.

Marketed TKI drugs are typically given continuously via the oral route and at a flat dose. Although a most effective and durable target saturation is the primary objective for dose development of TKI drugs, it is obvious that for several TKI drugs the recommended dose is the same as the reported MTD, e.g. Bosutinib, Pazopanib, Ponatinib or Sunitinib (Table 3). The dose-limiting toxicities include grade 3 gastrointestinal and hepatic toxicities, grade 3 skin toxicities, grade 3 fatigue, and grade 3 hypertension. For Sunitinib grade 2 bullous skin toxicity, grade 3 fatigue, and grade 3 hypertension are reported as dose-limiting toxicities. Furthermore, at approx. twice the therapeutic concentration a grade 2 QT-prolongation is expected (Summary of Product Characteristics/SmPC Sutent® [16]).

**Table 3:** Clinical pharmakokinetic profiles of TKI marketed in the EU

**TKI**	**t**_**max **_**(h)**	**Bioavailability (oral, %)**	**Concomitant food intake effect on bioavailability**	**Concomitant food intake: FDA recommendation**	**V (L/kg) 70-kg subject assumed**	**Primary enzymes involved in metabolism**	**Major metabolites**	**Plasma half-life (h)**	**Plasma protein binding (%)**	**Suggested threshold for response or concentration attained in therapy (mg/L)**
Bosutinib	6	18 [20] derived from colon tumor xenograft models		With food	131-214 [21]	CYP3A4	M2 (oxydechlorinated Bosutinib) M5 (N-desmethyl Bosutinib)		94-96	
Dasatinib	0.5–3	<34	Increases AUC (14%)	With/without food	30-40	CYP3A4, FMO-3	M4 (BMS-582691), M5 (BMS-606181), M6 (BMS-573188)	3–5	92–97	0.01–0.1 [22]
Erlotinib	4	69-76	Increases bioavailability (24%–31%)	Without food	3	CYP3A4, CYP3A5, CYP1A2	NorErlotinib (OSI-420)	41	92-95	>0.5
Gefitinib	3-7	57	No effect	With/without food	24	CYP3A4, CYP2D6, CYP3A5 (possibly CYP1A1)	NorGefitinib (M523595)	48	79	>0.2
Imatinib	2–4	98	No effect	With food	2–6 (Imatinib), 15–40 (NorImatinib)	CYP3A4, CYP3A5, CYP2C8	NorImatinib (CGP74588)	12–20 (Imatinib), 40–74 (NorImatinib)	95 (Imatinib and NorImatinib)	>1 (CML and GIST)
Lapatinib	3-5	-	Increases AUC (167%–325%)	Without food	31	CYP3A4, CYP3A5	Norlapatinib (GW690006)	14	>99	>0.5 mean concentration in patients prescribed 1500 mg once daily [23]
Nilotinib	3	30	Increases C_max_ (112%) and AUC (82%)	Without food	10–15	CYP3A4, CYP2C8	-	15–17	98	>0.6 C_min_ concentration applicable to quartile 1 from cytogenetic response [24]
Pazopanib	2.8	14-39	Increases AUC and C_max_ (2-fold)	Without food	0.1-0.2	CYP3A4, CYP1A2, CYP2C8	Pazopanib M24, Pazopanib M26, Pazopanib M27	31	>99	>20
Ponatinib				With/without food		CYP3A4 (MRI PI)	inactive carboxylic acid		>99	
Sorafenib	2-14	<50	Reduces bioavailability (29%)	Without food	3-6	CYP3A4, UGT1A9	Norsorafenib, Sorafenib N-oxide (BAY 67 3472)	20-40	>99	>3
Sunitinib	6-12	-	No effect	With/without food	30	CYP3A4	Norsunitinib (SU12662)	40–60 (Sunitinib), 80–110 (Norsunitinib)	95 (Sunitinib), 90 (Norsunitinib)	>0.05 (Sunitinib + Norsunitinib)
**TKI**	**DLT**	**MTD**	**Clinical dose (as recommended by SmPC)**	**Dosage form**	Human AUC at the clinical dose (ng*h/ml)	**In vitro IC**_**50 **_**values for target kinase inhibitor (ng/ml)**	**Dose-reduction**			
							**Liver**		**renal**	
Bosutinib	Grade 3 diarrhea, grade 3 rash [25]	500 mg, q.d	500 mg, q.d.	Tablet	2740 ± 790	250 nM [26]			Yes	
Dasatinib	Grade 3 nausea, grade 3 fatigue, grade 3 rash [27]	>120 mg b.i.d	100 mg, q.d. (for chronic phase), 70 mg, b.i.d. (for accelerated phase and blast phase)	Tablet	398.8 (b.i.d. regimen)	0.0976	No, only in severe liver impairment		No	
Erlotinib	Diarrhea [28]	150 mg, q.d.	150 mg, q.d.	Tablet	42679	0.787 [29]	No		No	
Gefitinib	Nausea, diarrhea, vomiting, rash	700 mg, q.d.	250 mg, q.d.	Tablet	7251.5	12.1 [30]	No, only in severe liver impairment		No	
Imatinib	Nausea, vomiting, fatigue, diarrhea	>1000 mg, b.i.d.	400 mg, q.d	Tablet	33200	12.3 [31]	Yes		No	
Lapatinib	Rash, diarrhea, fatigue	1800 mg, q.d.	1250 mg, q.d.	Tablet	33836.5	6.02 [32]	Yes		No, only in severe renal impairment	
Nilotinib	Liver function abnormalities, thrombocytopenia [33]	600 mg, b.i.d.	400 mg, b.i.d. (for chronic-phase and accelerated-phase of chronic myelogenous leukemia), 300 mg, b.i.d. (for newly diagnosed chronic-phase myelogenous leukemia)	Capsule	19000 (b.i.d. regimen)	not available	No		No	
Pazopanib	Grade 3 aspartate aminotransferase (AST)/alanine aminotransferase (ALT) elevations, grade 3 malaise [34]	800 mg, q.d. [35,36]	800 mg, q.d.	Tablet	650 ± 500 μg*h/ml	10, 30, 47, 71, 84 or 74 nM	Yes		No	
Ponatinib	Rash, fatigue	45 mg, q.d	45 mg, q.d.	Tablet	77 (50%) or 1296 (48%)	0.4 or 2.0 nM	Yes		No	
Sorafenib	Hand-foot skin syndrome (HFS) [37]	600 mg, b.i.d.	400 mg, b.i.d.	Tablet	36690 (b.i.d. regimen)	7.79 [38]	No		No	
Sunitinib	Grade 3 fatigue, grade 3 hypertension, grade 2 bullous skin toxicity (HFS) [39]	50 mg, q.d.	50 mg, q.d.	Capsule	1406	0.797	No, only in severe liver impairment		No	

AUC, area under the curve; b.i.d., twice daily; DLT, dose limiting toxicity; MTD, maximum tolerated dose; q.d., every day; tmax, time after administration when Cmax is reached; Source of information: Summaries of Product Characteristics (SmPCs) of marketed TKI [16] unless otherwise indicated.

From a clinical point of view there are arguments for consideration as an NTID for selective TKI which are elucidated for the example of Sunitinib: The dose of 50 mg/d is the recommended dose for renal cell carcinoma and the MTD at the same time. The documented adverse events (AE) and adverse drug reactions (ADR) are serious, and toxicity may be difficult to control due to long half-life of parent compound and main metabolite (40-60 h and 80-110 h, respectively). The described toxicity induces a high probability of dose reductions with the intent to reduce exposure. The patient safety may be impaired in case of an exchange between originator and generic medicinal product following dose reduction: Dose reductions of 12.5 mg represent a 25% and 33% decrease from the recommended dose for renal cell carcinoma and neuroendocrine tumors of pancreatic origin, respectively. In case of exchange of the originator for a generic drug the AUC from the reduced dose of the generic may be the same as the AUC from the normal dose of the originator if normal acceptance criteria for BE (90% CI for AUC and C_max_ 80-125%) are applied.

From a safety point of view it should be mentioned that chronic exposure to a dose that was identified as the maximum tolerable dose in a short term study may render the tolerable short term toxicity into intolerable long term toxicity.

### Safety of certain TKI

#### Dasatinib, Nilotinib & Bosutinib – CML-TKI with different safety profiles from a regulatory point of view and availability of second generation TKI

In general TKI are well tolerated in clinical practice, particularly, if compared with the toxicity of cytostatic drugs normally used in oncology. Often side-effects are only mild (grade 2 and lower) and occur early in the treatment course. Frequently they last only some days or weeks and resolve spontaneously. Moreover, even if drug-related toxicity requires drug discontinuation, re-exposition is often successful and permanent dose reduction is rarely necessary.

The advent of Imatinib in 2001 has dramatically changed the prognosis in patients with chronic myeloid leukemia (CML): The five year survival rate of patients with chronic phase CML improved from approximately 20% in the pre-TKI era to more than 90% patients [17]. In those patients who achieve a stable cytogenetic response with Imatinib overall survival is reported with 95.2% at 8 years in the literature and thus does not differ statistically significantly from that of the general population [18]. Imatinib is still the most common TKI modality used as a frontline therapy in CML across the world. However, due to the occurrence of Imatinib resistance and intolerance, second generation TKI as Dasatinib, Nilotinib and Bosutinib have been developed. In non-clinical models they are 30 to 300 times more potent than Imatinib and can inhibit most Imatinib-resistant BCR-ABL mutations (EPARs for Imatinib, Dasatinib, and Nilotinib [15]). Comparable with the experience in anti-infective drugs, multidrug-resistant BCR/ABL mutations occur which preclude further use of the approved TKI. For example, patients with T315I mutation respond only on treatment with third generation TKI Ponatinib, which was specifically designed as a treatment option for these populations.

TKI indicated in CML have some side-effects in common as myelosuppression, gastrointestinal complaints, rash, fatigue, headache and peripheral and periorbital edema; however, intensity varies significantly between the different products. Other AE are peculiar of each drug: Imatinib has been uncommonly associated with severe heart failure, while Nilotinib is associated with QT prolongation, pancreatitis, increased rate of cardiovascular events, and occurrence of peripheral arterial occlusive disease (PAOD). Dasatinib may cause pleural, pericardial and peritoneal effusions; additionally interaction with platelet function is discussed to explain higher rates of gastrointestinal bleeding observed in clinical practice. Bosutinib is associated with significant gastrointestinal toxicity (diarrhea) and hepatotoxicity. Serious AE observed with Ponatinib are an alarming high rate of arterial thrombosis, and cardiovascular events as well as hepatotoxicity.

Differences in the safety profiles of these TKI seem to be at least partially explained by the additional inhibition of other signaling pathways apart BCR-ABL [c-Kit, Src family kinases, PDGFR, and others].

However, it should be kept in mind that TKI treatment of CML has to be administered lifelong and knowledge about potential long-term risks and efficacy, especially for the second generation TKI Dasatinib, Nilotinib and Bosutinib, is still limited. Whether risks associated with Ponatinib treatment can be tolerated is currently under discussion again.

Not only from a regulatory perspective careful attention on recommended risk minimization measures as defined in the product information is at the end essential to avoid treatment complications that may completely jeopardize the sought treatment success.

### Orphan drug status of TKI

The orphan regulation aims at fostering drug development for serious or life-threatening diseases with a prevalence of less than 5 in 10.000 people in the EU. A sponsor may apply for orphan designation any time prior to an application for marketing authorization (usually even before clinical development). The orphan drug status then needs to be confirmed during the marketing authorization procedure. The most important incentive of the regulation is ten year market exclusivity for an orphan medicinal product with respect to similar medicinal products. Neither EMA nor EU member states can authorize a product, which is regarded similar with respect to chemical structure and mode of action and therapeutic indication. Generics, by definition, fulfill all of these criteria.

Imatinib is the paradigm of targeted therapy with its target, the Philadelphia chromosome, occurring in two rare forms of cancer, CML and acute lymphatic leukemia (ALL) which remain rare in spite of recent advances for treatment. Other cancers, e.g. renal cell carcinoma, was recently reported to exceed the prevalence threshold of 5 in 10.000 people so that no further orphan designations are expected.

### Orphan similarity and market exclusivity

In addition to the incentive of the a.m. ten year market exclusivity intended by the European orphan regulation [19] there may be a probably unintended additional incentive. Special circumstances are conceivable under which the market exclusivity granted for orphan products may exclude marketing authorization of a generic product. These special circumstances first occurred when the orphan drug Tasigna® (Nilotinib) was assessed as “similar” to Glivec® (Imatinib). Glivec® was first authorized in the EU in 2003. The Committee for Medicinal Products for Human Use (CHMP) gave a positive opinion on its benefit risk balance, the Committee for Orphan Medicinal Products (COMP) confirmed the significant benefit and so Glivec® got the most important incentive for the development of medicines for orphan diseases – the market exclusivity. Under the condition of the European orphan drug regulation no medicinal product “similar” to Glivec® would get marketing authorization for ten years – unless the similar product had superior efficacy or safety or the MAH of the protected product gives consent to the marketing of the similar product.

Several years after marketing authorization of Glivec® was granted, similarity assessment of Tasigna® concluded that Tasigna® was a similar product to Glivec® and the market exclusivity of Glivec® would therefore be prohibitive for the authorization of Tasigna®. In the context of a similarity assessment, three characteristics of a given drug are decisive:

1) The chemical structure (respectively structural similarity to the innovator product)

2) The molecular mechanism of action, and

3) The indication(s).

In the first step of Tasigna® marketing authorization, this was not problematic, because Tasigna® was first authorized in second line after first line-therapy with Glivec®. However, with the extension of indications to first-line treatment of CML, Tasigna® was authorized only with the consent of the MAH of Glivec® (not surprisingly, as both medicines are products of Novartis). The COMP confirmed a significant benefit and thus Tasigna® received its ten own year market exclusivity beginning with the commission decision in 2007.

When data protection and orphan market exclusivity expired for Glivec® generic Imatinib products to the reference product Glivec® were submitted. There was, however, the previous regulatory decision that Glivec® and Tasigna® are similar products – including the assessment of Imatinib and Nilotinib as similar active substances based on their chemical structure and pharmacological mechanism. An authorization of a generic Imatinib product to the reference product Glivec® would therefore not be granted if it violated the 10 year market exclusivity of Tasigna® which began in 2007.

It is safe to assume that the European orphan legislation was never meant to preclude the authorization of generics after the data protection and the ten years orphan protection of the reference product had expired. And it also seems that this was not a deliberate abuse of a complicated legal and regulatory situation by Novartis but rather unintended. If that had been a wicked, albeit brilliant, marketing-driven strategy, the exact alignment of the indications of Glivec® and Tasigna® would have effectively prevented any Imatinib generics for many years. As the indications of Tasigna® and Glivec® overlap for the majority of patients but are not identical, a marketing authorization for Imatinib generics restricted to the indications not granted for Tasigna® became possible. This is why the indications of generic Imatinib products are different from the indications of the reference product Glivec®.

## Conclusion

A decade ago, TKI were introduced into clinical anti-cancer therapy. At first sight, the molecular mechanism of action appears to comprise only a targeted approach in blocking tyrosine kinases. However, this should not be misleading; numerous closely interconnected signaling pathways are involved and the complexity of TKI molecular mechanism is far from being understood completely. For clinicians, TKI are a worthy new modality of tumor-therapy amending classical cytotoxic regimes. TKI are of substantial benefit in terms of efficacy with a tolerable safety profile. However, long-term safety issues might not be fully elucidated at present and, thus, cannot be finally judged upon. Throughout the next years, many of these substances will run off-patent. Thus, regulatory guidance will be required for instance on whether certain substances like Sunitinib fulfill the criteria of a *narrow therapeutic index drug*. Apart from that, most TKI are orally administered, thereby raising the question whether BCS-based biowaiver can apply. In addition, design and requirements of BE-studies will be an issue in the EMA-initiative of product specific guidance on anti-cancer-TKI.

## Abbreviations

ADR: Adverse drug reaction; AUC: Area under the curve; ALL: Acute lymphatic leukemia; BCS: Biopharmaceutics Classification System; BE: Bioequivalence; b.i.d.: twice daily; CMA: Conditional marketing; Cmax: maximal plasma concentration; CML: Chronic myeloic leukemia; CHMP: Committee for medicinal products for human use; COMP: Committee for orphan medicinal products; CYP: Cytochrome P450; DLT: Dose limiting toxicity; EMA: European medicines agency; EPAR: European Public Assessment Report; FDA: U.S. Food and Drug Administration; GIST: Gastrointestinal stromal tumor; HFS: Hand foot syndrome; MAA: Marketing authorization applications; MEC: Minimum effective concentrations; MTC: Minimum toxic concentrations; MTD: Maximal tolerated dose; NTID: Narrow therapeutic index drug; NSCLC: Non-small cell lung cancer; PAOD: Peripheral arterial occlusive disease; PK: Pharmakokinetic; pNET: pancreatic neuroendocrine tumors; q.d.: every day; RTK: Receptor tyrosine kinase; STS: Soft tissue sarcoma; TKI: Tyrosine kinase inhibitor tmax, time after administration when Cmax is reached.

## Competing interests

The authors declare that they have no competing interests.

## Authors’ contributions

All authors filed the manuscript, NE and LR performed a systematic search on clinical PK-parameter. All authors read and approved the final manuscript.
